# SR 11302, an AP-1 Inhibitor, Reduces Metastatic Lesion Formation in *Ex Vivo* 4D Lung Cancer Model

**DOI:** 10.1007/s12307-017-0202-0

**Published:** 2017-11-24

**Authors:** Dhruva Kumar Mishra, Min P. Kim

**Affiliations:** 1 Department of Surgery, Houston Methodist Research Institute, 6670 Bertner Ave, Houston, TX 77030 USA; 2 Department of Surgery, Weill Cornell Medical College, Houston Methodist Hospital, Houston, TX 77030 USA

**Keywords:** Lung cancer, Metastasis, AP1, SR11302, 4D lung model

## Abstract

Activator protein (AP) -1 is a transcription factor, plays important role in cell differentiation, proliferation and apoptosis. Analysis of tumor cells grown on *ex vivo* 4D lung cancer model shows increase in components of AP-1, c-Fos and c-Jun in circulating tumor cells (CTC) compared to primary tumor. Our aim was to determine whether the AP-1 inhibitor SR11302 reduces metastatic lesion formation in the 4D model. Human lung cancer cell lines A549, H1299, and H460 were grown in the 4D model and treated with SR11302 (1 μM). We compared the number of cells in the metastatic site upon SR11302 treatment and number of viable CTCs isolated from the 4D model with parental cells treated/untreated with SR11302 on a petri dish. There were significantly fewer tumor cells per high-power field on metastatic site in 4D model seeded with H460 (*p* = 0.009), A549 (*p* = 0.01), or H1299 (*p* = 0.02) cells treated with SR11302. Furthermore, the CTCs from SR11302 treated 4D models, seeded with H460 (*p* = 0.04), A549 (*p* = 0.008), or H1299 (*p* = 0.01) cells had significantly fewer viable tumor cells after 4 days in culture than the respective untreated control. However, the SR11302 had no impact on the viability of parental H460 (*p* = 0.87), A549 (*p* = 0.93), or H1299 (*p* = 0.25) cells grown on a petri dish (2D). SR11302 reduces metastatic lesion formation in the *ex vivo* 4D lung cancer model due to the presence of an independent yet common pathway among three cell lines. The *ex vivo* 4D model may provide a tool to better understand the complex process of metastasis.

## Introduction

Lung cancer is the leading cause of cancer-related death in the United States [1]. The predominant cause of lung cancer-related death is organ failure due to metastatic lung cancer to other organs. To better understand lung cancer progression and metastasis, we have developed a four-dimensional (4D) lung cancer *ex vivo* model, from acellular rat lung, which has intact basement membrane that allows tumor cells to grow in the epithelial space and continuous nutrient flow and perfusion to occur through the vascular space. It is different from the conventional 2D/monolayer cells which are grown on flat and rigid substrates and 3D cell culture [2] where cells grow in an artificial extracellular matrix component in three-dimensional fashion, however, there is absence of vasculature. We have named it as four-dimensional (4D) model as it provides an additional dimension of continuous controlled flow over the time along with conventional 3D growth of cells. It mimics the major steps of human lung cancer progression such as primary tumor nodules, circulatory tumor cells (CTCs) and metastatic lesion formation [3–5]. The 4D model also mimics MMPs secretion such as MMP-9 seen in patients with lung cancer that is not seen in the same cells that are grown on a petri dish (2D). Moreover, the gene signature of the primary tumor from the 4D model predicts poor survival in human lung cancer patients [6, 7]. Our previous gene expression analysis involved in tumor cells undergoing transformation from the primary tumor to CTCs to metastatic lesions in H1299 cells from the 4D model yielded several candidates that may be important in tumor metastasis [8]. Among those genes, we have found that, both c-Fos, and c-Jun, components of AP-1, are elevated in CTCs compared to primary tumors and metastatic lesions [8].

Recent studies have elucidated a crucial role for the AP-1 transcription factor in tumorigenesis [9–11]. The AP-1 transcription factor is a dimeric complex, mainly composed of c-Jun, c-Fos, and activating transcription factor (ATF) protein families. Jun family members (c-Jun, JunB, and JunD) can form a transcriptionally active homodimer; however, Fos and ATF family members need to heterodimerize with members of the Jun family to form a transcriptionally active complex [12–14]. AP-1 complexes bind to TPA response elements or cAMP response elements in the promoter regions of target genes and regulate the proliferation, differentiation, apoptosis and transformation of cells within the context of a complex dynamic network [15].

A group of natural or synthetic vitamin A analogues, such as Retinoids, are considered potential antitumor agents that mediate their effect through binding to different nuclear receptors such as the AP-1 complex [11, 16]. Clinical studies indicated that retinoic acid is effective for the treatment of certain types of leukemia and is a chemopreventive agent against the occurrence of secondary head and neck cancers [17]. After screening several synthetic retinoids, Fanjul and coworkers found that SR11302, a chemical compound, inhibits the AP-1 activity without activating the retinoic acid response elements [18, 19]. Therefore, in our current study, we hypothesize that SR11302 treatment may inhibit the metastatic tumor growth in a 4D *ex vivo* model seeded with lung cancer cell lines.

## Materials and Methods

### Animal Handling and Cell Culture

The animal experiment protocols were approved by the Institutional Animal Care and Use Committee at the Houston Methodist Research Institute. All experiments were carried out in accordance to guidelines and policies governing the use of laboratory animals in research. We obtained authenticated lung cancer cell lines A549, H1299, and H460 from American Type Culture Collection (ATCC, Manassas, VA, USA) and grew them on culture flask before seeding on the *ex vivo* 4D model. American Type Culture Collection (ATCC, Manassas, VA, USA) performed the verification of cell lines by DNA profiling. All the cell lines were maintained in RPMI1640 media (Gibco, USA) supplemented with 10% FBS (Gibco, USA) and 1X antibiotic (Gibco, USA).

### *Ex Vivo* 4D Lung Cancer Model

Lung and heart block was harvested from 6- to 8-week-old Sprague Dawley rats and decellularized using 0.1% sodium dodecyl sulfate followed by 1% Triton-X 100 [3–5]. Acellular lungs were set up in a customized bioreactor, connected with an oxygenator and pump for culture media perfusion through lung vasculature. These acellular lungs were placed in a bioreactor with the right main stem ligated with a silk suture to create a metastatic *ex vivo* 4D lung model as previously described [5]. Approximately 25 million A549, H1299, and H460 cells were seeded to the epithelial space of the lung through the trachea, populated in the left lung lobes or primary tumor, and incubated for 2 h in an incubator. Next, the bioreactor was connected to a pump and oxygenator and was run at 6 cc per min. Every day, 200 ml of culture media was replenished and the used media was centrifuged at 500 g for 5 min to count the number of CTCs. The experiment was carried out for 12–20 days and right lobectomies with metastatic lesions were performed on day 9–10 (right upper), day 12–15 (right middle) and day 15–20 (right lower).

### RNA Extraction and Real-Time PCR Analysis

Total RNA was extracted from the primary tumor, metastatic lesions, and CTCs using Direct-zol RNA miniPrep (Zymo Research, Irvine, CA, USA). RNA quality and quantity was determined using a Nanodrop 1000 spectrophotometer (Thermo Scientific, Waltham, MA, USA). cDNA was prepared using a high-capacity cDNA reverse transcription kit (Applied Biosystems, Grand Island, NY, USA) with 500 ng of total RNA and qPCR assay was performed with SYBR green reagent (Bioline, USA). We used the published 18S rRNA (18 s F – TTTTCGGAACTGAGGCCATG and 18 s R – CTTGGCAAATGCTTTCGCTC) as a housekeeping gene and primers were designed for the other target genes (c-Jun and c-Fos) (c-Fos F – CACTGCCATCTCGACCAGT, c-Fos R – TTCCTTTCCCTTCGGATTCT, c-Jun F – ACGCAAACCTCAGCAACTTC and c-Jun R - CACTGTCTGAGGCTCCTCCT). A relative fold of gene expression was calculated using 2^-(ΔΔCt)^ formula.

### Treatment of Lung Cancer Parental (2D) Cells with SR11302

To compare the effect of AP-1 inhibitor SR11302 (Santa Cruz Biotechnology, Dallas, TX, USA) on parental (2D) cells, we plated 100,000 human lung cancer cells per well (H1299, H460, or A549) in 2 mL of complete media on a 12-well culture plate and treated with (*n* = 3) or without (n = 3) SR11302. A stock solution of 10 mM was prepared in ethanol and aliquots were stored at −80 °C [18]. We added 1 μM of SR11302 to treated cells while the same volume of ethanol was added to the untreated control cells. After 4 days, we counted the number of live cells in culture after trypsinization, using the trypan blue method by TC20 Cell Counter (BioRad, Hercules, CA, USA). We compared the total number of live cells for each cell line treated with or without the inhibitor.

### Treatment of the 4D Model with SR11302

The *ex vivo* 4D metastatic lung cancer model was created as described above. The 4D lung cancer model was seeded with either A549 (*n* = 2), H1299 (*n* = 6), or H460 (n = 6). One of the pair was treated with 1 μM of SR11302 daily while the control was treated with an equal volume of ethanol. We obtained gross measurements of the primary tumor on day 5, day 10, day 15, and day 20. Primary tumor was preserved in 10% formalin and then hematoxylin and eosin (H&E) and immunohistochemistry for Ki-67 (proliferation index) was performed by Houston Methodist Research Institute Pathology Core as described before [4]. CTCs were collected and counted from treated and untreated bioreactors every day. To further check the viability of these CTCs, we plated 10,000–25,000 cells in a 96-well plate and counted the total number of live cells after 4 days of incubation in 200 μL of fresh culture media. Total and live cell counting was done using trypan blue staining with a TC20 automated cell counter (Bio-Rad).

Finally, lobectomy was performed on day 9–10, day 12–15, and day 15–20 of the right lung to assess the metastatic lesion. Primary tumors were evaluated on day 20. All the lobectomy tissues were preserved in 10% formalin and then H&E was performed. We then counted the number of metastatic lesions in ten random images in high-power field (40X) using Evos XL (Life Technologies, Carlsbad, CA, USA).

#### Cell Migration assay

We used the 8 μm, 24 transwell plate (Corning, USA) to determine the migration efficiency of cells. Transwells were seeded with equal number (100,000 cells/well) A549 and H1299 cells in in Serum free media in duplicate. Culture media with 10% serum was used as chemoattractant in the bottom of each well. In treatment group, 1 μM SR11302 was added. Plates were incubated for 16 h in cell culture incubator at 37 °C, 5% CO2 atmosphere. Next day, non-migrating cells were removed using cotton swab moistened with medium from top of the permeable support. Migrated cells on the lower surface were stained with 0.1% Crystal violet stain. It is further washed with distilled water thrice for approximately 2 min and then membrane is let air-dried. We took images under microscope at 10X power field and stain was further dissolve in 2% SDS for 30 min and absorbance was taken at 590 μm.

### Statistical Analysis

All analyses were performed using PRISM Version 5.0 software (GraphPad Software, La Jolla, CA, USA). Independent two-sample t-tests were performed to identify the significantly different mean number of live cells after drug treatment for CTCs and 2D cells. A Student’s t-test was performed to compare the c-Jun and c-Fos levels and the number of cells per high-power field between the groups. A *p* < 0.05 was considered to be statistically significant.

## Results

### C-Jun and c-Fos Were Upregulated in CTCs from *Ex Vivo* 4D Model as Compared to Primary Tumor

**Fig. 1:**
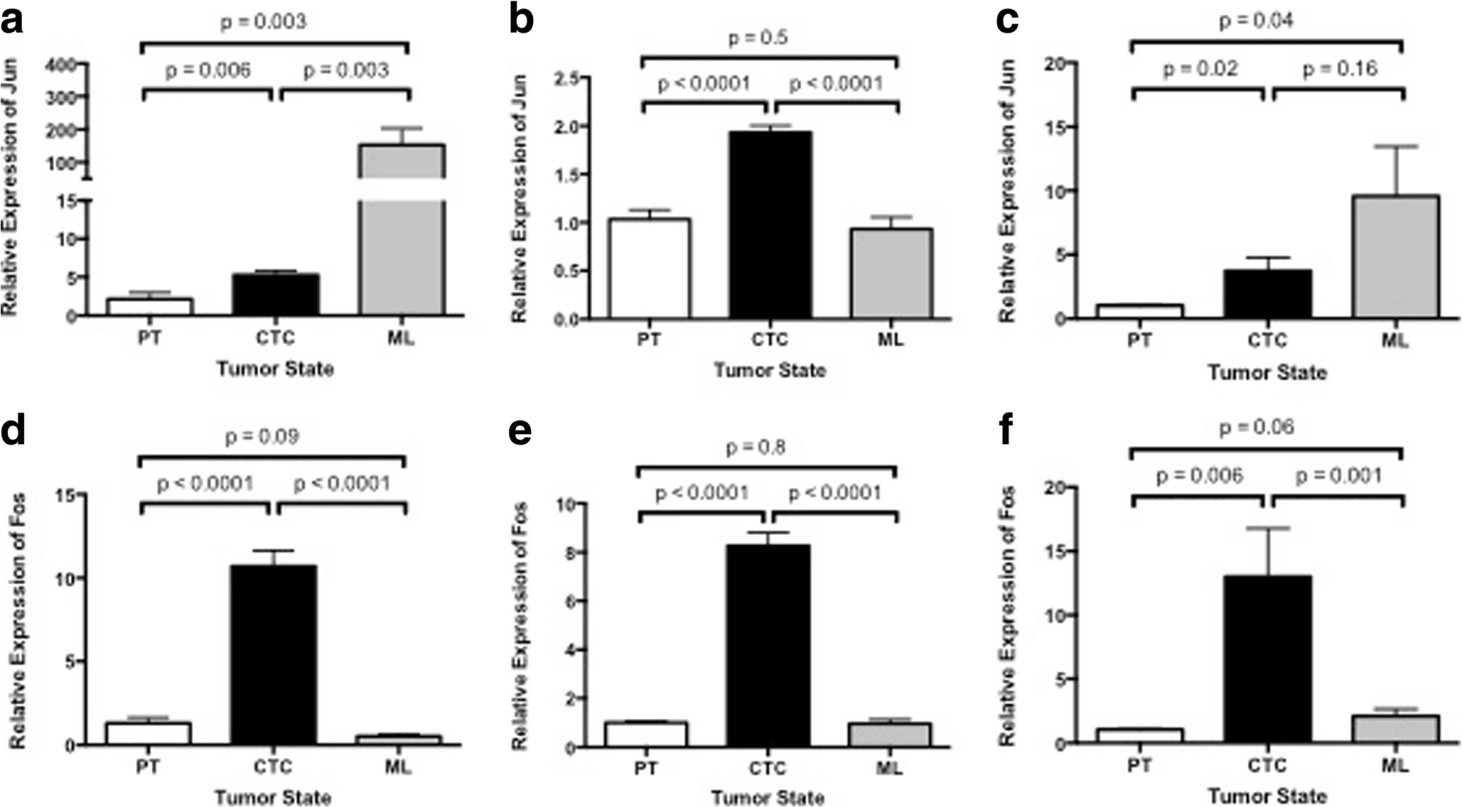
Relative gene expression (2^-ΔΔCt^) of c-Jun and c-Fos genes in primary tumors (PTs), circulatory tumor cells (CTCs), and metastatic lesions (MLs) from a 4D *ex vivo* lung model seeded with A549 (**a** & **d**), H1299 (**b** & **e**), and H460 (C & F), cells. **a, b,** & **c** c-Jun was significantly upregulated in CTCs as compared to respective PTs for all three-cell lines. **d, e** & **f**: c-Fos was significantly upregulated in CTCs as compared to respective PTs and metastatic lesions in all three-cell lines

Our gene expression analysis of c-Jun and c-Fos in the primary tumor, CTCs, and metastatic lesions of an *ex vivo* 4D model seeded with A549, H1299, and H460 cells showed a significant over-expression in CTCs compared to the primary tumor (Fig. 1). There was significantly increased expression of c-Jun in the CTCs compared to the primary tumor for A549 (Fig. 1a, *p* = 0.006), H1299 (Fig. 1b, *p* < 0.0001), and H460 (Fig. 1c, *p* = 0.02). The expression of c-Jun was significantly less in the metastatic lesion for H1299 compared to CTC (Fig. 1b *p* < 0.0001) while there was no difference for H460 (Fig. 1c, *p* = 0.16), and an increase for A549 (Fig. 1a, *p* = 0.003). The c-Fos expression was consistent through the different phases of tumor progression. The CTCs had significantly higher expression of c-Fos compared to primary tumors for A549 (Fig. 1d *p* < 0.0001), H1299 (Fig. 1e, *p* < 0.0001), and H460 (Fig. 1f, *p* = 0.006). Moreover, CTCs had significantly higher expression of c-Fos compared to metastatic lesions for A549 (Fig. 1d, *p* < 0.0001), H1299 (Fig. 1e, *p* < 0.0001), and H460 (Fig. 1f, *p* = 0.001).

### SR11302 Inhibits Metastatic Lesion Formation on 4D Lung Model

**Fig. 2:**
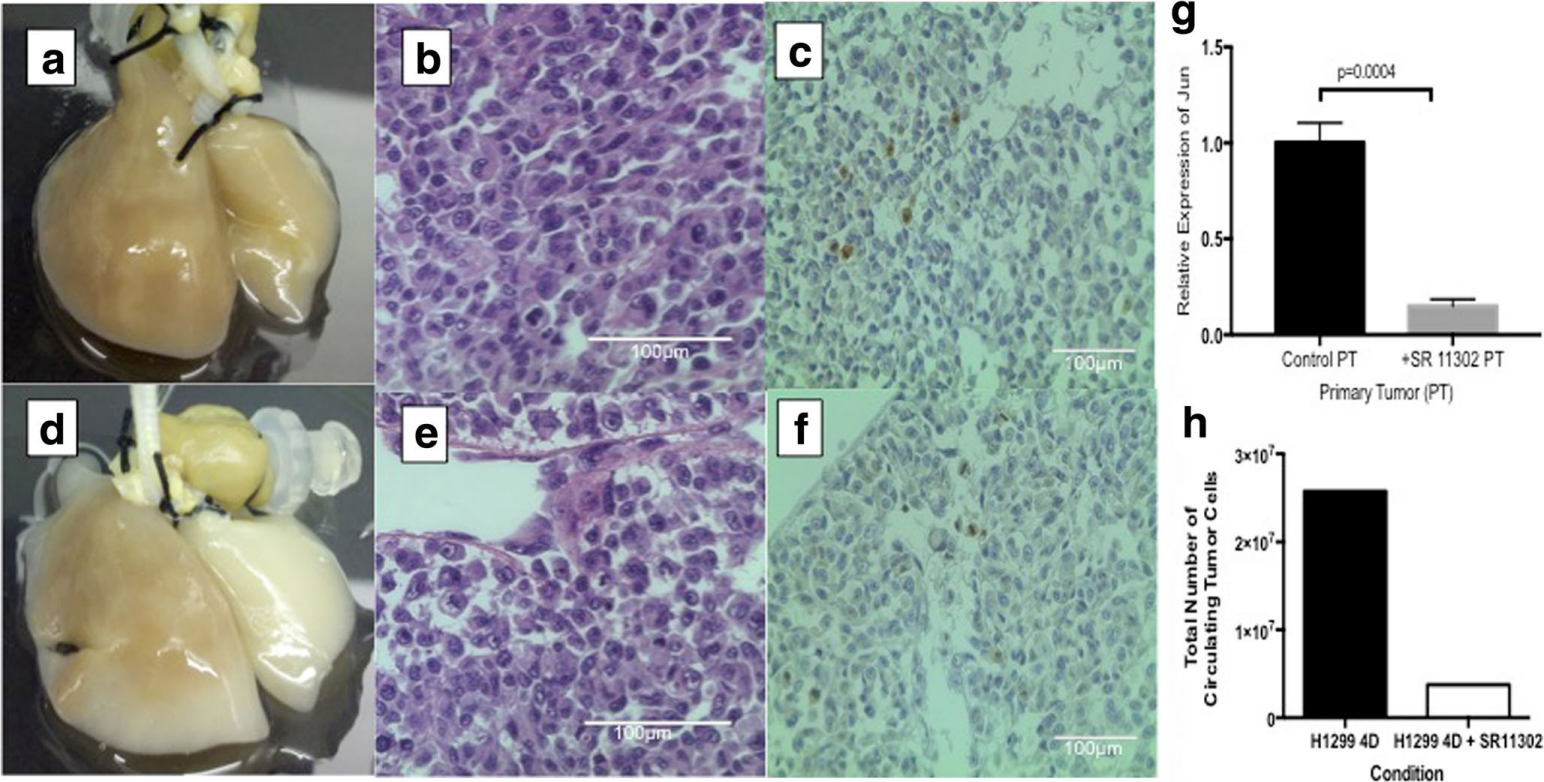
Effect of SR11302 on the primary tumor of ex vivo 4D lung model seeded with H1299 cells. **a** Image of the primary tumor (left) and metastatic lesion (right) on day 25 of the 4D model seeded with H1299 without treatment showing tumor (brown) on both sides. **d** Image of the primary tumor (left) and metastatic lesion (right) on day 25 of the 4D model seeded with H1299 treated with SR11302. There is lack of visible tumor on the metastatic lesion side. **b** & **e** Hematoxylin and eosin image of the primary tumor. There is no difference in the cell morphology between the untreated (B) and treated (E) primary tumor. **c** & **f** Immunohistochemitry stain for Ki-67. There is no difference in proliferation index between untreated (C) and treated (F) 4D model seeded with H1299 (*p* = 0.33). **g** Relative gene expression of c-jun in primary tumor (PT) was significantly downregulated upon SR 11302 treatment (*p* < 0.001). **h** Total number of CTCs was less after after SR11302 treatment in 20 day period of cell culture on 4D model

Primary tumor nodules were formed in the 4D lung model seeded with H1299, both in untreated controls (Fig. 2a-c) and treated with SR11302 (Fig. 2d-f). There were grossly visible tumor nodules on the left lung that grew over time. There was no gross difference of the size of the nodule and cell morphology between the two groups (Fig. 2a & d). There was no significant difference in the proliferation index (Ki-67) between the primary tumor treated and untreated with SR11302 (*p* = 0.33) (Fig. 2 c & f). There was significantly less c-Jun (*p* = 0.0004) gene expression in the primary tumor treated with SR11302 compared to untreated control (Fig. 2g). In triplicates for 4D model seeded with H1299, the total number of CTC is lower in the 4D model with SR11302 compared to untreated control (Fig. 2h).

**Fig. 3:**
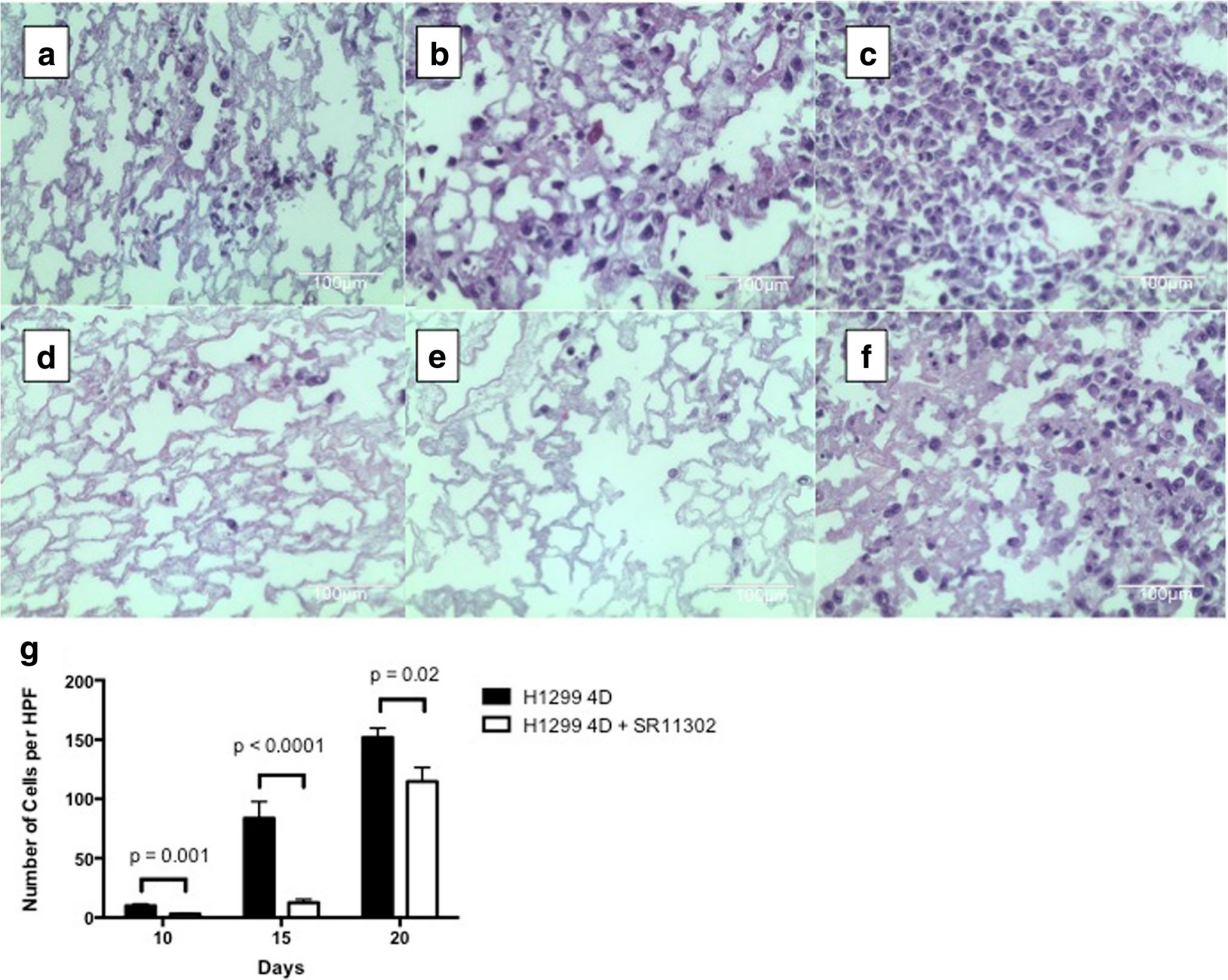
Effect of SR11302 on metastasis in ex vivo 4D model with H1299 cells: **a-f** hematoxylin and eosin images of right upper (A & D) on day 10, right middle (B & E) on day 15 and right lower (C & F) on day 20. The untreated control 4D lung has more metastatic lesion (A, B & C) compared to the SR11302-treated lung in 40X high power field image. **g** Graph shows significant difference in number of per high-power field (HPF) in metastatic lesion for 4D model seeded with H1299 compared to control on day 10 (*p* = 0.0001), day 15 (*p* < 0.0001) and day 20 (*p* = 0.02)

There was an increasing number of metastatic lesions over time in the untreated 4D model with H1299 starting with day 10 (Fig. 3a), to day 15 (Fig. 3b) and to day 20 (Fig. 3c) as well as the 4D model with H1299 treated with SR11302 starting with day 10 (Fig. 3d), to day 15 (Fig. 3e) and to day 20 (Fig. 3f). For each of those days, there were fewer metastatic lesions per high-power field for the 4D model treated with SR11302 on day 10 (Fig. 3g, *p* = 0.001), day 15 (*p* < 0.0001), and day 20 (*p* = 0.02) compared to the untreated control. We repeated this experiment for 15 days and it showed significantly less metastatic lesion formation on day 9 (*p* = 0.0003), day 12 (*p* = 0.0004) and day 15 (p < 0.0001) in the 4D model seeded with H1299 treated with SR11302 compared to control. This was also seen when the experiment was repeated again showing that there was significantly less metastatic lesion formation on day 10 (p = 0.0004) and day 15 (p < 0.0001) in the 4D model seeded with H1299 treated with SR11302 compared to control.

**Fig. 4:**
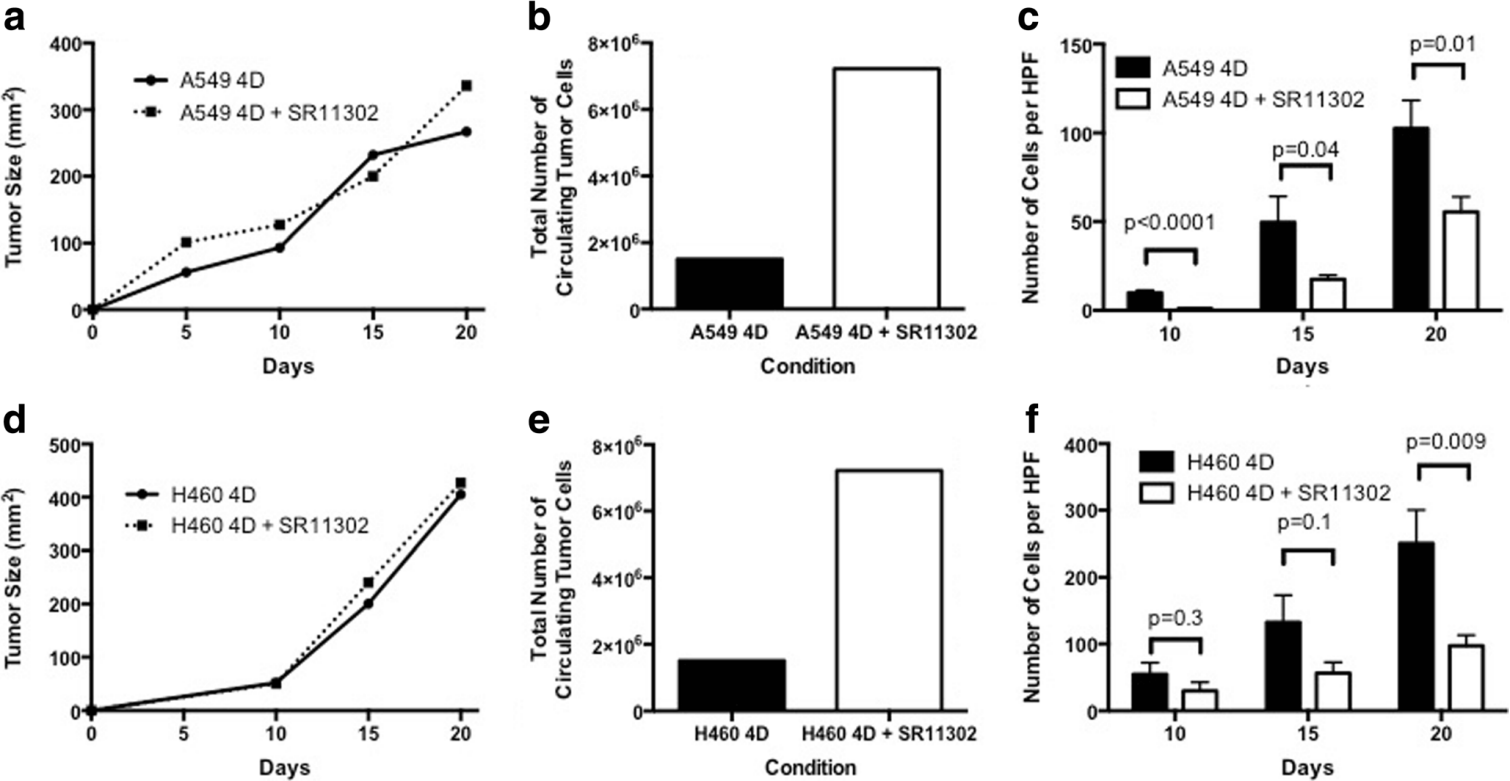
Effect of SR11302 on metastasis in ex vivo 4D model with A549 and H460 cells: No significant effect of SR11302 was observed on tumor nodule size with A549 (**a**) and H460 (**d**). SR 11302 treatment lead to the increase in total number of CTCs formed in 4D model seeded with A549 (**b**) and H460 (**e**). **c** There was significant inhibition of metastatic lesion formation in the 4D model seeded with A549 treated with SR11302 on day 10 (p < 0.0001), day 15 (*p* = 0.04) and day 20 (*p* = 0.01). **f** There is significantly less metastatic lesion on day 20 (*p* = 0.009) for the 4D model seeded with H460 treated with SR11302 compared to control

The primary tumor growth for the 4D model seeded with A549 did not differ with the treatment of SR11302 (Fig. 4a) with no significant difference in proliferation index (Ki-67) between treated and untreated control (*p* = 0.08). The total number of live CTCs for the duration of the experiment was higher for the 4D model treated with SR11302 compared to the untreated control (Fig. 4b). There were significantly fewer metastatic lesions per high-power field for the 4D model with A549 treated with SR11302 on day 10 (Fig. 4c, p < 0.001), day 15 (*p* = 0.04) and day 20 (*p* = 0.01) compared to the untreated control.

The primary tumor growth was not different for 4D model seeded with H460 and treated with SR11302 (Fig. 4d). Moreover, there was no significant difference in the proliferation index (Ki-67) between treated and untreated groups (*p* = 0.59). The total number of CTC varied in all three experiments (Fig. 4e). For two experiments there were lower number of total live CTC in the treatment group compared to the control while for one experiment there were higher number of total live CTC in the treatment group compared to control. For the 4D model seeded with H460, there was also increasing number of metastatic lesion with untreated on day 10, day 15 and day 20 and treatment with SR11302 on day 10, day 15 and day 20 (Fig. 4f). There was significantly less metastatic lesion with SR11302 on day 20 compared to control (*p* = 0.009). We repeated this experiment, which showed that there was significantly less metastatic lesion with treatment on day 10 (*p* = 0.01), day 15 (*p* = 0.006) and day 20 (*p* = 0.004, Fig. 4f). When we repeated the experiment for 15 day period, there was significantly less metastatic lesion with treatment on day 9 (*p* = 0.02) and day 15 (*p* = 0.007).

### Differential Impact of SR11302 at Different Phases of Tumor Development and Cell Migration

**Fig. 5:**
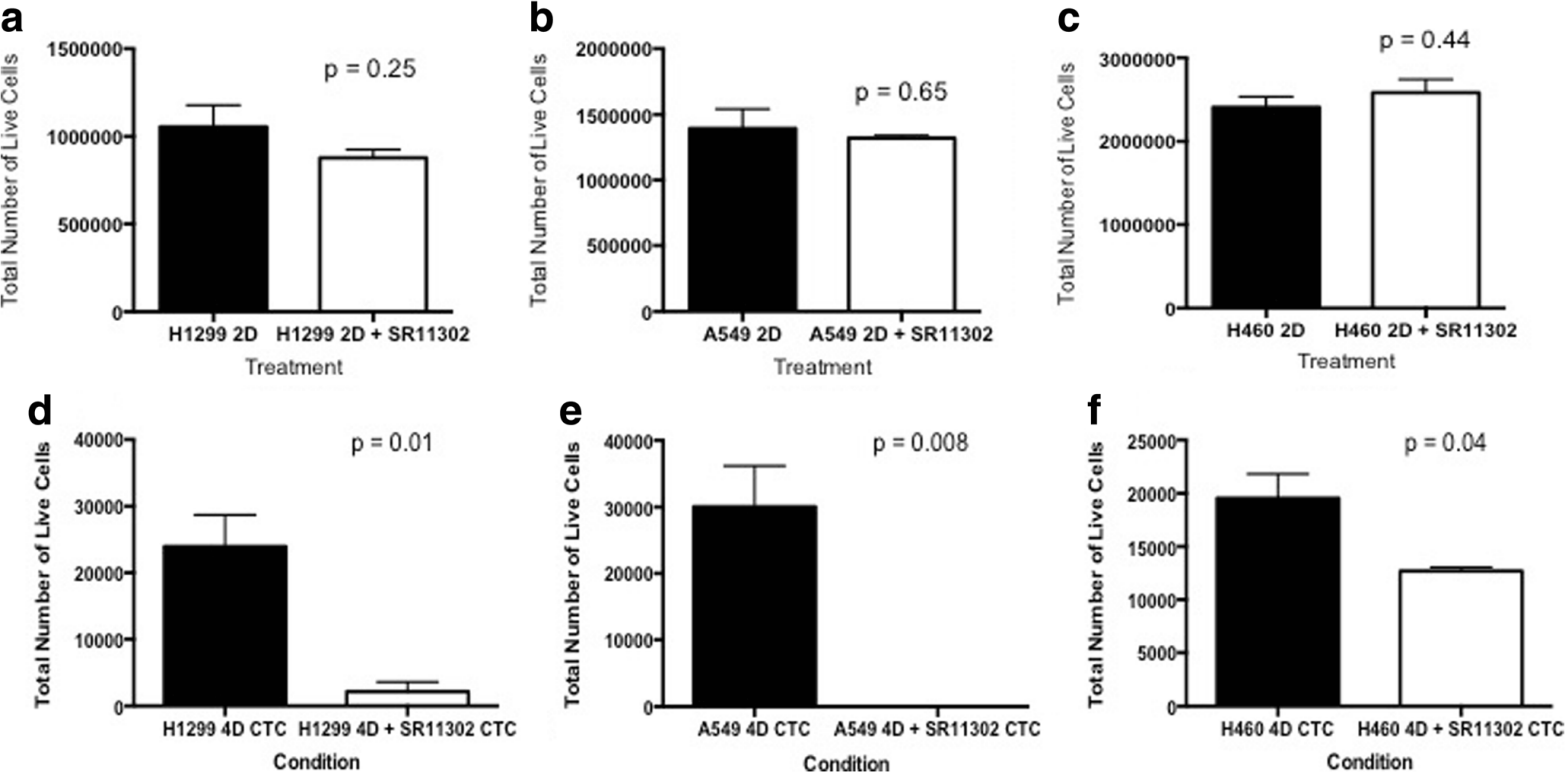
Differnetial impact of SR11302 at different points of tumor development: **a-c** There is no significant difference in the tumor number of cells that were grown on a petri dish (2D) when treated with SR11302 for 4 days for H1299 (*p* = 0.25, A), A549 (*p* = 0.65, B), and H460 (*p* = 0.44, C) cells. **d-f** When same number of CTC from the 4D model seeded with H1299 (D), A549 (E) or H460 (F) treated with or without SR11302 were plated on the petri dish for 4 days, there was significantly less viable cells with treatment for H1299 (p = 0.01), A549 (*p* = 0.008) and H460 (p = 0.04)

**Fig. 6:**
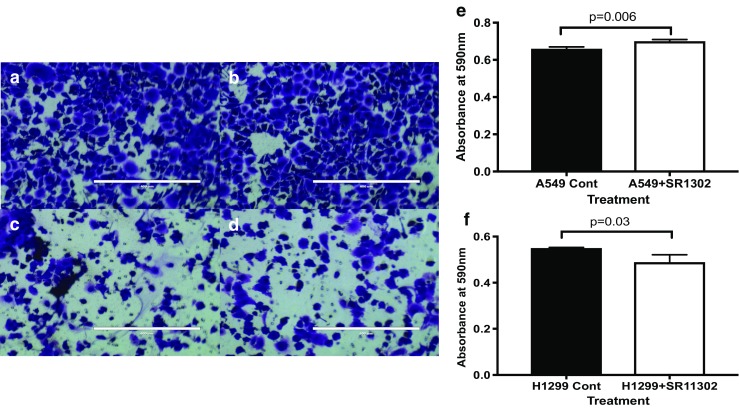
Impact of SR11302 on Cell migration of A549 and H1299 cells: **a, b** and **e** SR11302 treatment for 16 h significantly increases cell migration in A549 cells (B) as compared to untreated control (A). In contrast, cell migration significantly decreases upon SR11302 treatment (D) in comparison to untreated control with H1299 cells

SR11302 did not inhibit the growth of H1299 (Fig. 5a, *p* = 0.25), A549 (Fig. 5b, *p* = 0.65), and H460 (Fig. 5c, *p* = 0.44) on the petri dish (2D). However, when the same number of live CTCs was plated on a petri dish (2D) for 4 days, there were significantly fewer viable cells for CTCs from the 4D model with H1299 (Fig. 5d, *p*=0.01), A549 (Fig. 5h, *p* = 0.008) and H460 (Fig. 5f, *p* = 0.04) treated with SR11302 compared to the untreated control. SR11302 has a differential effect on A549 and H1299 cells. Upon treatment with 1μM SR11302, A549 showed a significant increase in cell migration (*p* = 0.006, Fig. 6a,b and e). However, H1299 cells showed the completely opposite behavior, as it was significantly decreased (*p* = 0.03, Fig. 6c, d and f).

## Discussion

Our results demonstrated that SR11302, an AP1 inhibitor reduces tumor metastasis in the 4D model by affecting the viability of CTCs without impact on primary tumor growth. This is the first time an *ex vivo* model can show an impact of a drug on metastasis without an impact in the primary tumor growth. As SR11302 is a known compound with strong anti-AP-1 activity with selective binding with RARα and RARϒ, but not with RARβ and RXRα [18], it provides important evidence that the AP-1 complex may play an important role in CTC survival and, ultimately, tumor metastasis in lung cancer.

Several studies suggested the crucial role of the AP-1 complex in cell transformation in which the activation of AP-1 mediates the pre-neoplastic to neoplastic progression [20–22]. Recent studies also showed its role in epithelial to mesenchymal transition, where AP-1 cooperates with the *Twist* gene and increases tumor invasiveness [23]. Different components of AP-1, such as Fra-1, induce changes in the expression of epithelial- to mesenchymal-transition-related transcription factors leading to the acquisition of mesenchymal, invasive, and tumorigenic capacities by epithelial cells [24]. In our study, we found the inhibition of metastatic lesion formation and decreased viability of CTCs upon AP-1 inhibitor treatment, suggesting that CTC viability in transit is impacted by AP-1 and with its inhibition more of the CTC undergo apoptosis and cannot form a metastatic lesion.

There was a variable number of total live CTCs with treatment of SR11302 in the 4D model without a clear pattern between the experiments. There may be two different explanation for this discrepancy. First, is that the concentration of SR11302 is not performing adequate inhibition when there is large number of CTC formation (H1299) compared to small number of CTC formation (H460 and A549). Second, is that counting the number of CTC after 24 h in the media may not reflect the true number of live CTC. Some of the live CTC that were counted may actually be in the process of undergoing apoptosis. Thus, when we count them 4 days later we find more of the cells that are truly live than the cells that were transiently live. Regardless of the number of CTCs produced, in all three-cell types, CTCs were less viable when plated on a petri dish and less likely to form metastatic lesions. A differential response of H1299 and A549 2D cells to cell migration upon SR11302 addition, may also contribute to variability in total live CTCs. Further mechanistic studies need to be performed to understand the role of AP-1 in the formation of CTCs.

Limitations of the study include the lack of molecular analysis to better understand the exact mechanism that leads to a decrease in cell viability of the CTC on a petri dish and the formation of the metastatic lesion in the 4D model. We plan to further characterize the exact mechanism of this phenotype by comparing the gene expression pattern of key genes upon SR11302 treatment. Another limitation of this study is the absence of other tumor microenvironment components such as lymphocytes, fibroblasts and endothelial cells. Furthermore, this study does not address the *in vivo* relevance. We plan to develop cell lines with inducible inhibition of AP-1, which can be used in *in vivo* metastatic mouse models of metastasis.

Overall, in this study we were able to show the antitumor effect of AP-1 inhibitor SR11302 on lung cancer metastasis and CTCs’ survival using an *ex vivo* 4D lung model. Our model can provide a better understanding of AP-1 regulators at the molecular level, as it provides an opportunity to analyze the cells in three major phases of tumor progression: primary tumor, CTCs, and metastatic lesion formation. Moreover, our study for the first time shows the impact of a drug in the reduction of metastatic lesion formation without inhibition of primary tumor growth in an *ex vivo* 4D model.
